# El reto de ganar credibilidad para poder innovar desde la provincia

**DOI:** 10.7705/biomedica.7111

**Published:** 2023-06-30

**Authors:** Iván Darío Vélez

**Affiliations:** 1 Fundador del Programa de Estudio y Control de Enfermedades Tropicales -PECET, Universidad de Antioquia, Medellín, Colombia, e investigador principal del World Mosquito Program en el valle de Aburrá, Antioquía, Colombia Universidad de Antioquia Universidad de Antioquia Medellín Colombia

*¿Qué sostiene el desarrollo económico en un mundo físico caracterizado por la disminución de los recursos y la escasez? La respuesta: la manera en que las sociedades tratan los avances tecnológicos.*

Paul M. Romer

Colombia es un país que invierte muy poco en investigación, menos del 0,3 % del producto interno bruto (PIB) por lo que está condenado a seguir en su dependencia tecnológica y al subdesarrollo.

Iniciativas esporádicas, temporales y focalizadas han tratado de cambiar este panorama y lograr, al menos, que alguna ciudad o región sirva de ejemplo y muestre que sí es posible investigar, innovar, transformarse y ser competitivos, independientemente de las aberrantes políticas nacionales donde la ciencia, la tecnología y la innovación son las cenicientas en las prioridades del Estado.

Ya hace más de 20 años que la llamada Primera Comisión de Sabios, luego de un trabajo juicioso, trazó un rumbo para que Colombia, por fin, fuera un país innovador y pidió que en pocos años la inversión en Ciencia y Tecnología fuera del orden del 3 % del PIB. Sin embargo, como se dijo, en el año 2023 está inversión no llega al 0,3 % con tendencia a disminuir en los próximos años.

En la primera década del presente siglo, Medellín creó un ecosistema de ciencia, tecnología e innovación, en parte como respuesta ciudadana a la época vergonzosa del narcotráfico y del sicariato, que llevó a esta ciudad a ser considerada como la más peligrosa del mundo.

Gracias al llamado de la Academia, en el 2003 se creó el Comité Universidad Empresa Estado y se tendieron puentes entre la universidad pública y la universidad privada; la universidad y la empresa; y la universidad, la empresa y el Estado en un trabajo armónico con las alcaldías y las gobernaciones qué duro 17 años, al menos en lo que se refiere a la participación de la Alcaldía de Medellín. Uno de los hitos importantes en esta alianza fue la creación de Ruta N en el año 2009: el Centro de Innovación y Negocios de Medellín, cuyo propósito es “contribuir al mejoramiento de la calidad de vida de los habitantes de la ciudad, a través de la ciencia, la tecnología y la innovación”.

En este ambiente de innovación se inscribe la puesta en marcha de una medida novedosa para combatir la principal enfermedad transmitida por vectores del valle de Aburrá que es el dengue, medida que implicaba la liberación de millones de zancudos portadores de una bacteria cuyo nombre prácticamente nadie conocía: *Wolbachia* spp.

Fue gracias a la invitación y al acompañamiento del *World Mosquito Program* que la Universidad de Antioquia, por medio del PECET y con el apoyo del Comité Universidad Empresa Estado y de la Ruta N, llevó a cabo este reto. Porque fue un reto conseguir la aprobación de las autoridades, de los comités de ética y de la sociedad en general, para liberar millones de *Aedes aegypti* infectados con la bacteria *Wolbachia* spp. en los territorios de las ciudades de Bello, Medellín e Itagüí, donde viven cerca de 3,5 millones de personas.

Para ello, inicialmente en el 2015 se llevó a cabo una prueba piloto en el barrio París de Bello, la cual demostró la aceptación voluntaria de la comunidad para la liberación de los mosquitos con *Wolbachia* spp. y el establecimiento de la bacteria entre los mosquitos locales, que ocho años más tarde persiste en niveles cercanos al 100 %.

Dadas las epidemias sucesivas de chikungunya (2014), zika (2015) y dengue (2016), todas transmitidas por A. aegypti, en América, se atendió el llamado de la OPS/OMS de evaluar, con ensayos controlados, la eficacia del control biológico con *Wolbachia* spp.

## ¿En qué consiste el control biológico con *Wolbachia* spp.?

Investigadores australianos encontraron que una bacteria exclusiva de insectos llamada *Wolbachia* spp. impide que los virus se reproduzcan en el mosquito. Lo interesante de *Wolbachia* spp. es que es una bacteria intracelular obligatoria, no vive por fuera de las células, no infecta otros seres diferentes y está ampliamente distribuida en los insectos del mundo.

Los australianos lograron transferir *Wolbachia* spp. de la mosca de las frutas al *A. aegypti*. La bacteria *Wolbachia* spp. solo se transmite por los huevos del mosquito e impide la transmisión, no solo del dengue sino también del virus del zika, del chikungunya, de la fiebre amarilla y de otros virus. Para controlar estas enfermedades se creó el World Mosquito Program.

Con el fin de lograr la aceptación comunitaria, el *World Mosquito Program* desarrolló el modelo de aceptación pública, una metodología transparente, ética y responsiva, para que la comunidad conozca los fundamentos del programa, participe del mismo, tenga rápidamente respuesta a todas sus inquietudes y decida voluntariamente sobre la liberación o no de los mosquitos con *Wolbachia* spp. en su barrio y ciudad.

El expandir las liberaciones a tres ciudades que suman más de 130 km^2^, implicó crear un insectario moderno que permitiera la producción de millones de mosquitos por semana. Insectario que, actualmente, prepara material para otros países de América.

La metodología implicó dividir las ciudades en cuadrados de 50 x 50 m y cada uno de estos fue un punto de liberación semanal de alrededor de 120 mosquitos, durante 20 semanas. Un trabajo arduo, coordinado y multidisciplinario.

Para evaluar la eficacia de *Wolbachia* spp. en la disminución de los casos de dengue, se inscribió el ensayo en el portal *Clinical Trials*, se publicó en una revista indexada y se incluyeron en el diseño del estudio, las series interrumpidas de tiempo: el comportamiento del dengue, el zika y el chikungunya, antes de las liberaciones y después ellas, de acuerdo con los registros de las secretarías de salud y del Instituto Nacional de Salud.

Además, se llevó a cabo un estudio de casos y controles (nivel de evidencia I) para lo cual se seleccionaron cuatro comunas de Medellín, hiperendémicas para dengue, con 500.000 habitantes, aproximadamente. En la mitad del territorio se liberaron mosquitos con *Wolbachia* spp. y la otra mitad sirvió de control.

Gracias al sistema de aseguramiento de salud que tiene la población colombiana, fue fácil establecer las clínicas y hospitales donde consultan las personas residentes en estas cuatro comunas de Medellín y se solicitó la colaboración de 11 de ella. A las muestras de casos sospechosos se les hizo reacción en cadena de la polimerasa (PCR) en tiempo real, para la amplificación de los virus de dengue, zika y chikungunya; presencia del gen NS1 para dengue y búsqueda de anticuerpos IgG e IgM. Posteriormente, los casos positivos se fueron ubicando en el mapa de acuerdo con su dirección de residencia.

Los resultados han sido extraordinarios. Las series de tiempo muestran disminución del 90 % de la incidencia de dengue en las tres ciudades, siendo la menor (89 %) en Medellín, donde por una decisión de la Secretaría de Salud, se fumigaron zonas donde se estaban liberando los mosquitos con *Wolbachia* spp. con el argumento de que, si había muchos Aedes, había riesgo de brote, ¡así no hubiera casos humanos!

Sin embargo, y a pesar de esto, desde el 2020, luego de haber terminado la liberación de los mosquitos con *Wolbachia* spp., Medellín ha presentado la menor tasa de incidencia y del número total de casos de los últimos 20 años. La última epidemia fue en 2016 con cerca de 17.000 casos y, desde el año pasado, Medellín debería estar en época de brote, pero en los primeros cuatro meses del 2023 el número de casos es menor a un centenar.

El estudio de casos y controles mostró que la incidencia de dengue fue un 70 a 73 % menor entre los participantes residentes en las áreas con mayor establecimiento de mosquitos con *Wolbachia* spp., en comparación con los participantes de los niveles más bajos de exposición.

Estos contundentes resultados están en poder de las autoridades de salud. Sin embargo, a pesar del incremento del dengue en el país, de no contar con otra herramienta efectiva, segura y sostenible, y de tener la capacidad instalada para expandir el control biológico a otras ciudades, desde la provincia se sigue a la espera de las decisiones políticas que se tomen en la capital para incluir el control biológico en el programa nacional de control del dengue. ¡Sin fundamento hay reticencia a innovar!

Debemos entender, como dijo Amparo Moraleda de IBM, que “La innovación es un desafío y no un drama, una oportunidad y no una amenaza”.
